# Cancer of unknown primary—Epidemiological trends and relevance of comprehensive genomic profiling

**DOI:** 10.1002/cam4.1689

**Published:** 2018-07-17

**Authors:** Carmen Binder, Katarina Luise Matthes, Dimitri Korol, Sabine Rohrmann, Holger Moch

**Affiliations:** ^1^ Division of Chronic Disease Epidemiology Epidemiology, Biostatistics and Prevention Institute University of Zurich Zurich Switzerland; ^2^ Department of Pathology and Molecular Pathology University Hospital Zurich Zurich Switzerland; ^3^ Cancer Registry Zurich and Zug University Hospital Zurich Zurich Switzerland

**Keywords:** cancer diagnostics, cancer of unknown primary, comprehensive genomic profiling, epidemiology, molecular profiling, next‐generation sequencing

## Abstract

**Background:**

Cancer of unknown primary (CUP) is a distinct clinicopathological entity with poor prognosis, frequently resistant to chemotherapy. Comprehensive genomic profiling (CGP) by next‐generation sequencing potentially identifies novel treatment options for CUP patients. The objective of this study was to determine incidence and survival trends and to discuss the value of CGP in CUP patients.

**Methods:**

Age‐standardized incidence rates (ASR) per 100 000 were calculated for 2935 CUP patients from 1981 to 2014 using cancer registry data of the canton of Zurich, Switzerland. Kaplan–Meier survival curves were estimated for sex, age, and histological groups. Cox proportional hazards regression models were used to estimate adjusted hazard ratios (HR). A literature review was conducted to assess the current use of CGP in CUP patients.

**Results:**

ASR of CUP increased from 10.3 to 17.6 between 1981 and 1997 and decreased to 5.8/100 000 in 2014. Mean overall survival remained stable. Mortality was significantly lower for patients with squamous cell carcinoma (HR 0.48 [95% CI, 0.41‐0.57]) and neuroendocrine carcinoma (0.75 [0.63‐0.88]) and higher for unclassified neoplasms (1.25 [1.13‐1.66]) compared to adenocarcinomas. The literature review identified 10 studies using CGP of CUP tissue. Clinically relevant mutations were identified in up to 85% of CUP patients, of which 13%‐64% may benefit from currently available drugs.

**Conclusions:**

CUP incidence decreased probably due to improved diagnostics, but mortality did not improve over the last 34 years. CGP testing may help to identify molecular signatures in CUP patients and enable targeted treatment.

## 1 INTRODUCTION

Cancer of unknown primary (CUP) is a heterogeneous group of aggressive metastatic tumors for which a standardized diagnostic workup fails to identify the site of origin at the time of presentation.^1^ It accounts for 2%‐5% of all new cancer diagnoses.^2^, ^3^, ^4^, ^5^ The inability to identify the tissue of origin in CUP patients is an immense clinical challenge, as the primary site of cancer influences treatment choices, outcome, and prognosis.^6^ Therefore, treatment options are limited in CUP patients and research efforts lag behind that of other solid tumor types.^7^ Conventional chemotherapy regimens, such as taxane based, platinum based, or combination of both, have not been able to substantially increase overall survival of unfavorable prognostic CUP groups.^8^ Therefore, these patients may present ideal candidates for personalized and targeted therapies.

The diagnostic workup of CUP includes the extensive use of diagnostic technologies, including modern imaging and endoscopy technologies on the one hand and detailed histopathological, immunohistochemical, molecular, and serum tumor marker investigations on the other. In general, tissue‐based diagnostics are considered a relatively cost‐efficient tool with substantial impact on diagnostic and therapeutic decisions.^9^ Recently, gene expression assays and next‐generation sequencing (NGS) have been proposed to determine the site of origin and potential treatment options in CUP.^10^, ^11^, ^12^, ^13^ Comprehensive Genomic Profiling (CGP) by NGS is a novel powerful tool to identify tumor‐specific genetic changes, which can be targeted with genotype‐directed treatment.^14^ For example, the majority (83%) of advanced breast and head and neck cancers, as well as melanoma patients harbored potentially actionable genetic alterations identified by NGS.^14^

Currently, CGP is introduced in many US and European laboratories. Due to poor prognosis and limited therapeutic options, CGP is a useful diagnostic approach especially in CUP patients. In this study, incidence and survival trends of CUPs were investigated using cancer registry data of the canton or Zurich. Furthermore, the current knowledge on CGP testing for the management of CUP patients was assessed by a literature review.

## 2 MATERIALS AND METHODS

### 2.1 Data source and study population

Cancer Registry of Zurich and Zug in Switzerland provided population data of the canton of Zurich for the period of 1981‐2014. This region is home to 18% of the Swiss population.^15^ Recent population‐based epidemiological studies consider different ranges of diagnostic codes for CUP patients from ICD‐O‐3 C80.9 only^16^, ^17^ to ranges ICD‐O‐3 C76/C77 to C80.9^4^, ^18^, ^19^ or even broader.^19^, ^20^ To avoid inclusion of patients with identifiable cancers that have been not accurately documented, only patients classified with a tumor of “unknown primary site” ICD‐10 code C80 were considered for this study, leading to a total number of 2935 patients. Further, 175 (5.9%) cases diagnosed on death certificate only and 91 (3.1%) cases diagnosed only by autopsy were excluded to analyze the time of survival after the initial diagnosis. This resulted in 2669 patients, which were included in the analysis. These patients were followed‐up until death or were censored on 31 December 2014, whichever came first. Patients were grouped into four histological and age subgroups. The histological groups were categorized as suggested by Fizazi et al^1^ based on the ICD‐O 3 morphological codes: adenocarcinomas (M‐814, M‐820, M‐821, M‐826, M‐831, M‐836, M‐843, M‐848, M‐849, M‐857, M‐898), squamous cell carcinomas (M‐805, M‐807, M‐812, M‐856), neuroendocrine carcinomas (M‐804, M‐824), unspecified carcinomas (NOS), incl. undifferentiated carcinomas (M‐801, M‐802, M‐803, M‐823), and unclassified neoplasms/tumors (M‐800). The age groups were formed according to the age at diagnosis: <60, 60‐69, 70‐79, and ≥80 years.

### 2.2 Statistical analysis

Age‐standardized incidence rates (ASR) were calculated as cases per 100 000 person years using the standardized European population.^21^ Kaplan–Meier survival curves were estimated for sex, age, and histological groups. Multivariate Cox proportional hazards models were used to estimate hazard ratios (HR) and 95% confidence intervals (CI) for sex, age groups, and histological groups. As the incidence year did not have an influence on overall survival, it was not included in the final analysis. All statistical analyses were performed using R Version 3.4.0.

### 2.3 Literature review on CGP and expression profiling

A literature review was conducted to assess the current view and recent scientific advances with regard to CGP and expression profiling for CUP patients. The search was conducted in PubMed on 31 May 2018 with following key words: “cancer of unknown primary (CUP) and genetic/genomic profiling,” “cancer of unknown primary (CUP) and next‐generation sequencing,” “cancer of unknown primary (CUP) and sequencing,” “cancer of unknown primary (CUP) and molecular profiling,” and “cancer of unknown primary (CUP) and gene expression profiling.” Peer‐reviewed publications in English language that documented new insights about gene panels testing for CUP patients were considered. Relevant references in the identified publications were also consulted.

## 3 RESULTS

### 3.1 Patient population, trends in incidence, age at diagnosis and survival

**Figure 1:**
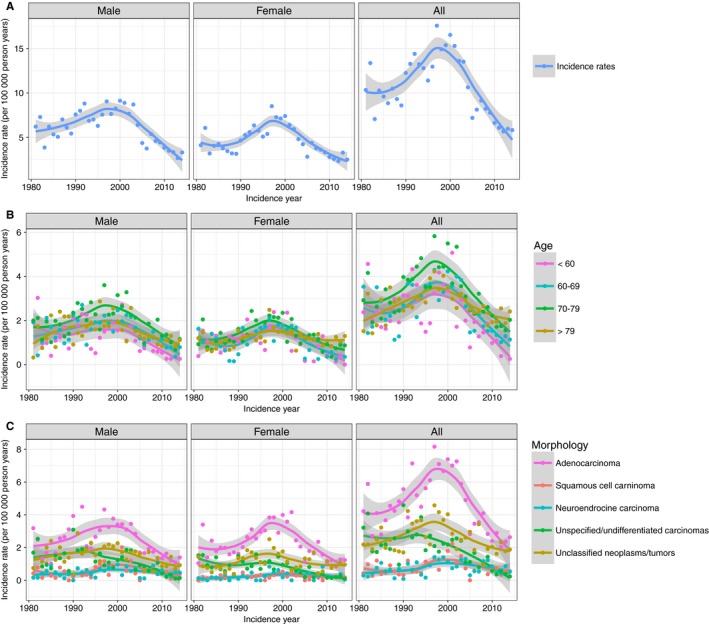
Age‐standardized incident rates of CUP in the canton of Zurich 1981‐2014. A, Overall, B, by morphology, C, by age group

There were 2935 patients registered with CUP in the canton of Zurich between 1981 and 2014, of which 47.0% were male and 52.0% were female. Mean age at diagnosis was 72.9 years (SD ±12.7; median = 75.0). The largest histological group was adenocarcinomas (42.3%), followed by unclassified neoplasms/tumors (28.1%) and unspecified carcinomas (NOS), incl. undifferentiated carcinoma (16.6%). Squamous cell carcinomas and neuroendocrine carcinomas accounted for <7% of the cases each (Table 1). CUPs accounted for 0.9%‐2.6% of all new cancer diagnoses with a peak in 1997 and with a decrease to 1.1% in 2014 (Figure S1). Similarly, there was an increasing trend of ASR from 10.3/100 000 person years in 1981 to 17.6 in 1997. After the tipping point in 1997, the ASR constantly decreased, reaching the lowest rate of 5.8/100 000 in 2014 (Figure 1). ASR were similar in males and females (Figure 1).

**Table 1:** Characteristics and survival time of CUP patients in the canton of Zurich

			All	Adenocarcinoma	Squamous Cell Carcinoma	Neuroendocrine carcinoma	Unspecified carcinomas	Unclassified neoplasms
Patients	Total	N (%)	2935 (100.0)	1241 (42.3)	193 (6.6)	188 (6.4)	488 (16.6)	825 (28.1)
	Men	N (%)	1380 (47.0)	538 (43.4)	129 (66.8)	108 (57.4)	259 (53.1)	346 (41.9)
	Women	N (%)	1555 (53.0)	703 (56.6)	64 (33.2)	80 (42.6)	229 (46.9)	479 (58.1)
Age in years	Total	Mean ± SD	72.9 ± 12.7	70.7 ± 12.4	69.3 ± 13.0	67.5 ± 13.1	70.1 ± 12.8	80.0 ± 9.9
		Median	75.0	73.0	69.0	69.0	72.0	82.0
	Men	Mean ± SD	71.2 ± 12.3	69.5 ± 11.4	68.8 ± 12.4	66.1 ± 12.7	68.9 ± 13.1	77.9 ± 9.9
		Median	73.0	71.0	69.0	68.5	71.0	80.0
	Women	Mean ± SD	74.5 ± 12.9	71.6 ± 13.1	70.3 ± 14.1	69.4 ± 13.5	71.6 ± 12.3	81.5 ± 9.5
		Median	77.0	74.0	69.5	72.0	73.0	84.0
Age groups	<60	N (%)	474 (16.1)	247 (19.9)	41 (21.3)	44 (23.4)	109 (22.3)	33 (4.0)
	60‐69	N (%)	540 (18.4)	254 (20.5)	57 (29.5)	51 (27.1)	101 (20.7)	77 (9.4)
	70‐79	N (%)	882 (30.1)	408 (32.8)	51 (26.4)	58 (30.9)	151 (30.9)	214 (25.9)
	>80	N (%)	1039 (35.4)	332 (26.8)	44 (22.8)	35 (18.6)	127 (26.0)	501 (60.7)
Months of Survival	Total	Mean ± SD	12.3 ± 31.6	10.8 ± 29.2	31.0 ± 54.5	19.6 ± 37.2	13.3 ± 34.5	6.7 ± 17.6
		Median (Q1‐Q3)	2.9 (0.9‐9.0)	3.0 (1.0‐8.3)	10.1 (3.1‐26.5)	5.0 (1.5‐21.8)	3.4 (1.1‐9.8)	1.4 (0.5‐4.5)
	Men	Mean ± SD	11.3 ± 29.1	9.3 ± 26.1	22.3 ± 42.2	21.4 ± 45.3	12.1 ± 29.0	6.1 ± 15.6
		Median (Q1‐Q3)	2.7 (0.9‐8.3)	2.6 (0.9‐7.3)	8.3 (3.1‐22.3)	3.7 (1.1‐16.8)	3.2 (1.2‐9.7)	1.3 (0.4‐4.5)
	Women	Mean ± SD	13.1 ± 33.7	12.0 ± 31.4	47.8 ± 69.9	17.5 ± 23.5	14.7 ± 39.9	7.2 ± 19.1
		Median (Q1‐Q3)	3.0 (0.9‐13.1)	2.6 (0.9‐7.3)	16.5 (3.7‐53.5)	3.7 (1.1‐16.8)	3.5 (0.9‐10.2)	1.4 (0.5‐4.5)

N = number, % = in percentage SD = standard deviation, Q1 = quartile 1, Q3 = quartile 3.

**Figure 2:**
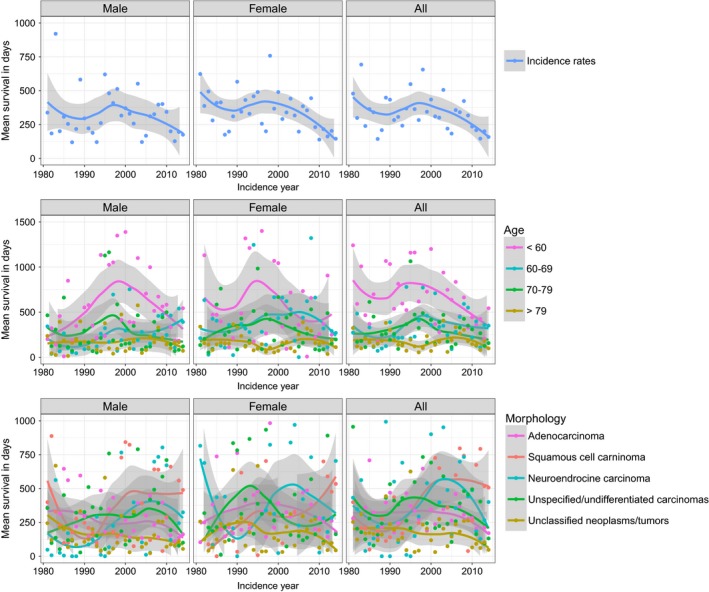
Mean survival in days of CUP patients in the canton of Zurich 1981‐2014. A, Overall, B, by morphology, C, by age group

**Figure 3:**
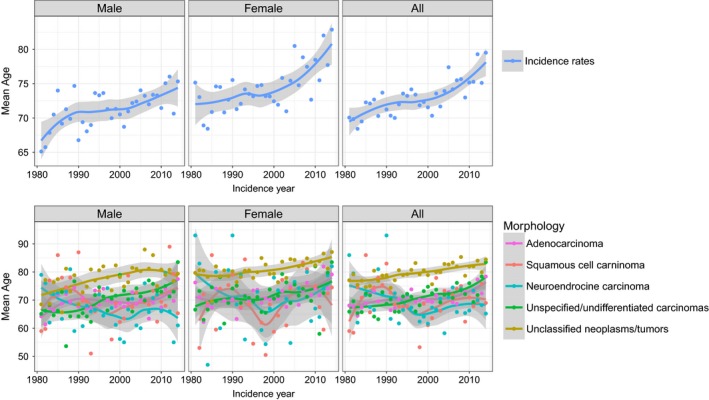
Mean age at diagnosis for CUP patients in the canton of Zurich 1981‐2014. A, Overall, B, by morphology

**Figure 4:**
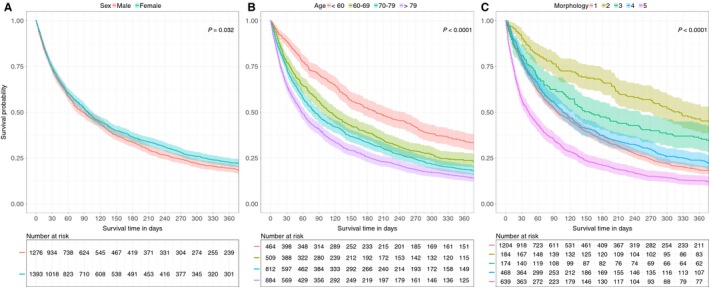
Kaplan–Meier survival curves for death in CUP patients depending on sex, age and morphology A, overall, B, by age group, C, by morphology with 1: adenocarcinomas, 2: squamous cell carcinomas, 3: neuroendocrine carcinomas, 4: unspecified/undifferentiated carcinomas, 5: unclassified neoplasms/tumors

Mean survival time after diagnosis was 12.3 ± 31.6 months (median 2.9). The average survival time after diagnosis has not improved from 1981 to 2014 (Figure 2). However, there was an increase in CUP patients’ age from 70 years in 1981 to 80 years in 2014 (Figure 3). The mortality was significantly higher in patients with unclassified tumors and neoplasms (HR 1.25 [95% CI 1.13‐1.66]) compared to adenocarcinomas. Squamous cell carcinomas (HR 0.48 [95% CI 0.41‐0.57]) and neuroendocrine carcinomas (HR 0.75 [95% CI 0.63‐0.88]) showed a significantly lower mortality compared to the adenocarcinoma (Table 2). Patients under 60 years showed a significantly lower mortality compared to patients with 60‐69 (HR 1.45 [95% CI 1.27‐1.66]), 70‐79 (HR 1.60 [95% CI 1.41‐1.81]) and above 80 years of age (HR 1.87 [95% CI 1.65‐2.12]). Women had a lower mortality compared to men (HR 0.85 [95% CI 0.78‐0.92]). The above‐identified factors are reflected in the Kaplan–Meier survival curves stratified by sex, age, and histological groups (Figure 4).

**Table 2:** Mortality of CUP patients adjusted for sex, age and histological groups

	HR	Lower CI	Upper CI
Sex			
Male	1.00 (ref.)	1.00 (ref.)	1.00 (ref.)
Female	0.85	0.78	0.92
Histology			
Adenocarcinoma	1.00 (ref.)	1.00 (ref.)	1.00 (ref.)
Squamous cell carcinoma	0.48	0.41	0.57
Neuroendocrine carcinoma	0.75	0.63	0.88
Unspecified/undifferentiated carcinomas	0.90	0.81	1.01
Unclassified neoplasms/tumors	1.25	1.13	1.66
Age			
<60	1.00 (ref.)	1.00 (ref.)	1.00 (ref.)
60‐69	1.45	1.27	1.66
70‐79	1.60	1.41	1.81
>80	1.87	1.65	2.12

HR, hazard ratio; CI, 95% confidence interval.

### 3.2 Comprehensive genomic profiling and expression profiling

Several reviews address the potential of NGS.^22^, ^23^, ^24^ However, so far only 10 published studies documented the application of NGS on tissue or blood samples of CUP patients. Eight studies reported CGP of CUP patient cohorts with varying sizes (16‐1806 patients or samples), whereas two studies documented individual case reports (Table 3). Mutations with potential therapeutic relevance that could affect therapy selection were identified in 30%‐85% of CUP patients, depending on the definition.^12^, ^13^, ^25^, ^26^, ^27^ In a recent study by Varghese et al^27^, who defined actionable alterations as biomarkers that are linked to a drug response either by FDA approval or other strong clinical evidence, targetable genomic alterations were observed through CGP in 30% of the patients. In a study by Kato et al^26^, CGP allowed for identifying clinically relevant genomic alterations potentially targetable by FDA‐approved drugs in 63.8% of the patients, whereas 1.6% of the patients had alterations targetable with agents under investigation in clinical trials. This led to a total of 65% of the patients harboring potentially actionable alterations in the named study, which was the only one to use liquid biopsies for CGP of CUP syndromes.^26^ In a previous study by Ross et al^13^, as many as 85% of the patients had genomic alterations that could potentially affect treatment decisions, but only for 13% of the patients these alterations were associated with approved targeted therapies. The remaining were linked to registered clinical trials and could enable patient entry into such.^13^ In all cases, neither immunohistochemistry, fluorescence in situ hybridization, serum biomarker analysis nor mRNA transcriptional profiling had led to further insights.^13^ There were significant differences in the genetic profile of adenocarcinoma CUP compared to nonadenocarcinoma CUP, suggesting differing targeted therapeutic strategies for these different CUP types.^13^ In general, most CUP patients harbor unique molecular profiles.^26^, ^28^ The results of Löffler et al^29^ identified genomic alterations in 84% of patients. About 15% of them included approved drugs. Gatalica et al^25^ used a multi‐platform profiling (including immunohistochemistry, gene sequencing and in situ hybridization). Whereas this group identified only a limited number of genetic mutations by CGP, various protein markers enabled the identification of druggable targets in 96% of the cases.^25^ A recent study selectively assessing predictive biomarkers for immune checkpoint inhibitors observed that 28% of the assessed CUP patients would have been potentially eligible for such therapies.^30^ In addition to these larger cohorts of CUP patients, two case studies illustrated the potential benefit of CUP patients from targeted therapies after genetic testing with immune checkpoint and mTOR inhibitors, respectively^31^, ^32^ (Table 3).

Our literature review also revealed 31 publications describing gene expression or epigenetic profiling in CUP patients using either PCR or microarray based assays (Table S1). Most studies aimed to demonstrate that gene expression profiling complements standard pathologic evaluation in determining the tissue of origin in CUP using commercially available or custom made assays.^33^, ^34^, ^35^, ^36^, ^37^, ^38^ Sample size varied greatly from 16 to 262 samples suitable for analysis per study.^34^, ^39^ Success rate of commercialized assays identifying the tissue of origin ranged from 61% to 98%.^38^, ^39^, ^40^, ^41^ The assay applied in the study with the lowest success rate of 61% identified the molecular profile of six tumor types based on 10 gene markers.^40^ On the other hand, in a study applying a commercially available 92‐gene assay, 247 of 252 (98%) patients had a tissue of origin predicted.^39^ A total of 194 patients then received assay‐directed site‐specific treatment subsequently leading to a median survival time of 12.5 months, which is favorable compared to previous results with empiric chemotherapy.^39^ With a distinct assay quantitating 48 miRNAs, 62 of 74 cases (84%) the result was consistent with the clinicopathologic picture.^35^ In seven cases, a tissue of origin was predicted that was not classified with conventional diagnostics.^35^ Two studies performing epigenetic profiling with a DNA methylation microarray on CUP tissue samples, showed a prediction accuracy of 78% and 87% for a primary tumor, respectively.^42^, ^43^ Furthermore, patients receiving subsequent tumor‐specific therapy showed a threefold improved overall survival compared to patients receiving empiric therapy.^42^ More details can be found in Table S1.

**Table 3:** Summary of publications on oncogene panel testing for CUP patients

	First author	Year	Number of genes	Techno‐logy	Panel vendor	Sample size	Results
Retrospective or prospective trials	Gatalica et al^30^	2018	592	NGS, IHC	Caris	389	About 28% of patients potentially eligible for immune checkpoint inhibitors when assessed with the following biomarkers: high tumor mutational load (46 of 389 patients [11.8%]), high microsatellite instability (7 of 389 [1.8%]), and PD‐L1 expression (82 of 365 patients [22.5%])
	Varghese et al^27^	2017	341‐410	NGS	MSK	150	Forty‐five of 150 patients (30%) showed targetable genomic alterations, which are either biomarkers with FDA approval or with otherwise strong clinical evidence to link to drug response. Furthermore, 38 patients (25%)
	Subbiah et al^28^	2017	255	NGS	FMI	17	Fifteen of 17 patients (88%) with genomic alterations, unique profile each. 11 (65%) patients were treated with a regimen that included a targeted therapy agent. Best responses were stable diseases for 4+ mo observed in four patients
	Kato et al^26^	2017	54‐70	NGS	Guardant Health	442	A total of 353 of 442 (80%) with genomic alterations. 289 (65%) with potentially targetable alterations. A total of 282 (63.8%) targetable off‐label with FDA‐approved drugs, 7 (1.6%) in clinical trials
	Löffler et al^29^	2016	50	NGS	‐	55	Forty‐six of 55 patients with genomic alterations (84%). Eight cases (15%) with mutations that may be targeted by currently approved drugs
	Ross et al^13^	2015	287	NGS	FMI	200	A total of 169 of 200 (85%) patients with clinically relevant genomic alterations. 26 (13%) alterations associated with approved targeted therapies, the rest linked to registered clinical trials
	Gatalica et al^25^	2014	47	NGS, IHC, in situ hybridiz.	‐	1806	In 96% of the cases, a biomarker associated with drug benefit was identified but mainly through established protein markers. Sequencing only detected a limited variety of genetic mutations
	Tothill et al^12^	2013	701	NGS	‐	16	Twelve of 16 samples with therapeutically relevant mutations (75%)
Case studies	Gröschel et al^31^	2016	WES	NGS	‐	1	Immune checkpoint inhibitor therapy with anti‐PD1 monoclonal antibody pembrolizumab resulted in rapid clinical improvement and lasting partial remission
	Wei et al^32^	2015	341 and 315	NGS	FMI, MSK	2	Mutations and clinicopathological features suggested clear‐cell renal cell carcinoma despite wild‐type VHL. Patients responded to mTORC1 inhibition therapy temsirolimus

NGS, next‐generation sequencing; WES, whole exome sequencing; IHC, immunohistochemistry; FMI, Foundation Medicine; MSK, Memorial Sloan Kettering.

## 4 DISCUSSION

Our study shows a decreased incidence of CUP patients, whereas prognosis of patients with CUP has not improved in the last 30 years. In the period from 1981 to 2014, CUP patients account for 0.9%‐2.6% of all newly diagnosed cancer patients in Zurich. This is in line with earlier studies in other Swiss regions identifying CUPs as 2.3% of new cancer diagnoses.^5^ CUPs account also for 2% of all new cancer diagnoses in the US,^5^, ^16^ but are about 4% in the Netherlands, South Australia, and Scotland.^4^, ^5^, ^16^, ^23^, ^44^ Differences between countries may be explained by different coding rules in cancer registries or different clinical or diagnostic procedures. In addition, reimbursement in the United States is more favorable for specific cancers compared to CUP. This could indirectly influence the classification of CUP cases as a specific cancer based on the physician's best guess may be chosen, leading to an underestimation of CUP incidence.^17^ With 28.1%, we report a substantially higher percentage of unclassified neoplasms/tumors, whereas 16.6% unspecified carcinomas is much lower than expected according to a recently published classification.^1^ This may also be due to coding methodology with some unspecified carcinomas being declared as unclassified neoplasms/tumors.

ASR increased to a high in 1997 and significantly decreased thereafter. The higher ASR between 1981 and 1997 may be due to increased CUP awareness and more frequent use of imaging technologies. The subsequent decrease in ASR may be due to better imaging technologies including computer tomography, broader use of endoscopy, and better immunohistochemical tools in pathology, which has led to a significant reduction in diagnostic errors in general.^45^, ^46^ Hence, also a higher identification rate of primary tumors may be assumed. In this time period, extensive immunohistochemical antibody panels have been introduced, including estrogen and progesterone receptors, neuroendocrine markers, different cytokeratins, as well as group‐ or organ‐specific tumor markers, for example, prostate (PSA), breast (NYBR1), lung/thyroid (TTF1), and renal cancer‐specific markers (RCC).^47^ However, it is estimated that immunohistochemistry helps to pinpoint the tissue of origin in less than 30% of CUP cases.^48^ Comparable time trends with a peak in the late 1990s have been observed in the Swedish, Scottish, and Australian population.^6^, ^19^, ^44^ In the Norwegian, Finnish, and US population, the tipping point was before 1990,^6^, ^16^, ^17^ which may be due to an earlier use of more aggressive diagnostics. In Scotland and Australia, ASR comparable with Switzerland have been documented.^5^, ^19^ For the US, however, lower ASR of 4‐6.6/100 000 person years have been reported, whereas Sweden reported ASR of 6/100 000 with the peak at 8/100 000 person years.^6^, ^16^, ^17^

Overall survival of CUP patients in Zurich has not improved over the last 34 years, which is similar to Sweden and the United States.^16^, ^20^ One reason for this limited treatment success could be the higher patient age at diagnosis in the last years. Whether the prognosis is worse for CUP compared to metastatic cancers of known primary depends on the sites of the primary tumors and the metastasis.^49^ Patient management and overall survival have improved significantly over the last decades for some metastatic cancers such as ovarian, colorectal, and breast cancer.^20^, ^50^ In contrast, improved survival has been limited to specific locations of CUP such as peritoneum, pelvis, and nervous system.^20^ We observed a better prognosis for CUP with squamous cell and neuroendocrine carcinoma CUP compared to adenocarcinomas. This is in line with recent reports about a slightly improved prognosis of CUP patients with squamous cell carcinoma, which may partly be due to aggressive treatment of nodal positive squamous cell carcinoma.^20^, ^50^

The lack of any treatment progress in patients with CUP led us to perform a literature review on novel diagnostic tools. To date, CUPs are mainly investigated by immunohistochemistry. Recently, gene expression arrays have been added to the diagnostic armamentarium, while the use of next‐generation gene sequencing for CGP to search for therapeutic targets has just begun.^13^, ^22^, ^26^ According to our literature search, gene expression profiling could complement standard pathologic diagnostic workup in determining the organ of origin in patients with CUP, particularly when immunohistochemistry is inconclusive.^24^, ^36^, ^38^ However, the added value of gene expression analyses varies, because the accuracy of the identification of the tissue of origin by gene expression profiling depends on the sample quality compared to blood‐based CGP.^37^ Hence, in certain clinical situations samples may not meet quality control criteria for the test and tissues might be misclassified.^37^, ^38^, ^51^ The success rate of the test also depends on the number of tissues covered by the test and its robustness. One study showed that a commercial assay covering six tumor types, only identified the tissue of origin of 63 of 104 patients as for 41 patients, the molecular profiles were not specific for the tissue types detectable by the assay.^40^ There is currently no gold standard to assess gene expression profiling tests, and most of the sample sizes are still fairly small.^52^ Nevertheless, gene expression profiles are considered a valuable addition to the standard diagnostic approach to identify the tumor of origin.^52^ Epigenetic profiles may add an additional piece to the puzzle of identifying the molecular profile of CUP patients.^53^ By assessing the DNA methylation profile of CUP patients, 78%‐87% of the primary tumor were identified.^42^, ^43^ An advantage of this approach over gene expression profiling is the use of DNA, a material stable over time, and less reactive to external factors compared to RNA.^42^

However, CUP can harbor genetic traits distinct from tumors of known primaries that may be clinically relevant.^54^, ^55^ They do not simply lack a few key markers of differentiation, but rather have fundamentally distinct gene expression patterns.^54^, ^55^ The incidence of mutations in the MET oncogene, for example, was significantly higher in CUP than in tumors of known origin.^56^

All CGP studies identified in our literature research identified clinically relevant genomic alterations using panels of 47‐701 genes.^12^, ^13^, ^26^, ^27^, ^28^, ^29^, ^30^ These are strong indications that potentially actionable genetic alterations are present in CUPs. However, it is no surprise that the rate of clinically relevant mutations in CUPs is strongly dependent on (a) the definition of what is to be considered a clinically actionable alteration and (b) panel type and size. Five of the eight studies identified such mutations with NGS technologies in >65% of the samples, which in 13%‐64% of the cases may be targeted with currently approved therapies (off‐label).^12^, ^13^, ^26^, ^29^ Other mutations could allow for including patients into ongoing clinical trials.^12^, ^13^, ^26^ A recent study defining actionable alterations rather narrowly as biomarkers that are linked to a drug response either by FDA approval or other strong clinical evidence, identified targetable genomic alterations by CGP in 30% of the patients.^27^ However, if genetic alterations, for which preclinical evidence for a specific drug response exists, are included, additional genomic alterations were observed in another 38 of the 150 patients leading to a total of 55% with potentially targetable mutations.^27^ If only biomarkers for immune checkpoint inhibitors were assessed, relevant mutations were found in 28% of the patients.^30^ Another study reporting lower mutation rates in CUP patients, applied a panel consisting of as little as 47 genes; hence, in the end, most druggable targets were identified by conventional immunohistochemistry and CGP only refined some diagnoses.^25^ However, more recent studies applying panels of 50‐70 genes have also detected mutations in >80% of the investigated cases.^26^, ^29^ Hence, panel selection rather than panel size seems to influence the amount of targetable mutations detected and the contribution of CGP to the diagnostic workup of CUP patients.

Given poor prognosis and limited treatment options for patients with CUP, genomic profiling using NGS technologies may meet a clinical need. A large number of CUP patients could benefit from molecularly targeted therapies, because clinically relevant mutations are observed in 30%‐85% of the CUP patients. Treatment based on the site of origin may be increasingly replaced by a treatment based on the molecular signature of the cancer leading to more precise and effective therapy.^54^, ^55^, ^57^, ^58^ Furthermore, dynamic change in the genomic profile of the tumor can be monitored and the treatment adapted accordingly.^26^

## 5 CONCLUSION

CUP remains an important clinical problem given frequency, outcome, and progress in a chemoresistant fashion. This study shows that the overall survival of CUP patients has not improved since the early 1980s. The reduced costs of NGS testing in the future and potential use of targeting agents for CUP could lead to increasing use of NGS, particularly if identification of the primary site becomes less relevant than the molecular signature of a CUP. Further development of personalized oncology for CUP will require clinical trials based on CGP.

## CONFLICT OF INTEREST

H.M. is an investigator in the F. Hoffmann‐La Roche Ltd study MX39795. He received honoraria from Roche. The other authors declare no conflict of interest.
