# Supplementary material for: Cancer of unknown primary—Epidemiological trends and relevance of comprehensive genomic profiling
Source: Cancer Med. 2018 Jul 17;7(9):4814–24. doi: 10.1002/cam4.1689 (PMC6144156; doi:10.1002/cam4.1689)
Supplement: Supplementary file 2 [file CAM4-7-4814-s002.docx]

**Supplement Table 1: Publications of PCR and microarray based analysis of CUP patients**

| **First author** | **Year** | **Type** | **Panel size** | **Technology** | **Sample size (for CUP / validation)** | **Results** |
| --- | --- | --- | --- | --- | --- | --- |
| Greco et al.^1^ | 2018 | Retrospective analysis of records | 92 | RT-PCR | 539 | 24 of 539 patients were diagnosed with renal cell carcinoma (4.4%) through molecular cancer profiling. None had suspected renal lesions. 20 of these received RCC specific treatment and had a median survival of 16 months. |
| Moran et al.^2^ | 2016 | Retrospective analysis of tissue samples | 850 | DNA microarray (DNA methylation profiles) | 216 | Prediction of primary site of origin in 188 of 216 (87%) of the cases with tumor type classifier EPICUP. |
| Greco et al.^3^ | 2015 | Retrospective analysis of tissue samples | 92 | qRT-PCR, mRNA expression profiling | 30 | 25 of 30 (83 %) patients diagnosed. 7 patients received site-specific therapy based on the diagnosis and remained alive from 25 to 72 and more months (5 progression-free). |
| Tothill et al.^4^ | 2015 | Prospective analysis of CUP samples | 29285 | mRNA microarray | 49 | Prediction of correct primary site in 38 of 49 CUP cases (78%). The classifier improved the accuracy of histology alone for both single and multiple site predictions and could predict the site of origin where histology alone could not suggest any specific diagnosis. |
| Pentheroudakis et al.^5^ | 2014 | Retrospective analysis of tissue samples | 5 | ddPCR | 87 | Proof of principle for evaluation of therapeutic strategies targeting pro-metastatic pathways in CUP patients as activating mutations were observed in KRAS, BRAF, PIK3CA, MET, and CTNNB1. MET and / or CTNNB1 activating mutations were prognostic for poor progression-free survival. |
| Kurahashi et al.^6^ | 2013 | Retrospective analysis of tissue samples | 5645 | RNA microarray | 60 | Correct identification of 26 of 30 samples (86.7%) as "CUPs". 237 genes were found to be either up-regulated or down-regulated by more than 2-fold for CUP samples of which 59 genes were of statistically significance as having biological attributes of CUP potentially contributing to a metastatic phenotype as well as resistance to anticancer drugs. |
| Greco et al.^7^ | 2013 | Analysis of tissue samples in parallel with standard pathologic evaluation | 92 | qRT-PCR for mRNA | 171 | High level of accuracy with identified latent primaries (75%), single IHC diagnoses (77%), and additional directed IHC and/or clinical/histologic findings (74%). |
| Hainsworth et al.^8^ | 2013 | Prospective trial | 92 | qRT-PCR for mRNA | 252 | 247 of 252 (98%) had a tissue of origin predicted. 194 patients received assay-directed site-specific treatment. In these 194 patients, the median survival time was 12.5 months hence favorable compared to previous results with empiric chemotherapy. Patients with treatment responsive tumor types and strong assay prediction even had a median survival time of 15.4 months. |
| Ades et al.^9^ | 2013 | Prospective analysis of CUP samples | 495 | mRNA microarray | 67 | Primary site identified with microarray test in 53 of 67 patients compared to 36 in case of clinical work-up. 31 patients with both clinical and gene expression diagnoses, matching in 11 cases (35%). |
| Tan et al.^10^ | 2013 | Case Report | 238 | PCR | 1 | Identification of EGFR mutation and initiation of gefitinib monotherapy led to significant shrinking of target lesions and a progression free survival of 11 months. |
| Fernandez et al.^11^ | 2012 | Retrospective analysis of tissue samples | 1322 | DNA microarray (DNA methylation profiles) | 42 | Assignment of a known tumor type in 29 of 42 cases (69%). Reconfirmation of this prediction for 7 of 9 cases (78%), for which blinded pathological analysis was developed at a later stage. |
| Meiri et al.^12^ | 2012 | Retrospective analysis of tissue samples | 64 | mRNA microarray | 52 | Clinical validation study on 52 CUP patients showed 88% concordance with the clinicopathological evaluation of the patients. |
| Ohta et al.^13^ | 2012 | Retrospective analysis of tissue samples | 3 | PCR | 9 | Mutation rate in CUP for codon 12 or 13 of the KRAS gene and for PIK3CA was lower than in colorectal cancer, while the same mutation rate for BRAF was almost the same. This means that the EGFR antibodies could possibly treat CUP. |
| Gross-Goupil et al.^14^ | 2012 | Prospective analysis | 495 | RNA microarray | 22 | Successfully performance of assay on 18 of 22 patients (82%). In the remaining 4 samples RNA preservation was too low for the test. |
| Hainsworth et al.^15^ | 2012 | Retrospective analysis of treatment outcome | 92 | RT-PCR | 42 | Patients predicted to have a colorectal site of origin by molecular tumor profiling treated with site-specific regimens showed median survival similar to patients with known metastatic colon cancer. The median survival in this group was substantially better than the historical median survival for patients with CUP (range 8-11 months) when treated with empirical CUP regimens. |
| Sorscher et al.^16^ | 2012 | Case Report | 92 | mRNA microarray | 1 | Diagnosis of papillary renal cell carcinoma, confirmed with additional immunohistochemical staining, allowed successful treatment with targeted agent (Everolimus). |
| Varadhachary et al.^17^ | 2011 | Prospective trial | 48 | qRT-PCR for miRNA | 74 | In 62 of 74 cases (84%) the assay result was consistent with the clinic pathologic picture. In 55 of the 65 where pathology and IHC suggested a diagnosis, the assay outcome was consistent (86%). For the 9 cases not classified with conventional pathology / IHC, the assay provided tissue of origin prediction compatible with the clinical presentation in 7 cases. |
| Ferracin et al.^18^ | 2011 | Retrospective analysis of tissue samples | 47 | RNA microarray | 16 | Predictions for probably primary site possible for all patient samples. For 12 samples the probability was >90% with low error rate suggesting highly reliable predictions. |
| Dumur et al.^19^ | 2011 | Retrospective analysis of tissue samples | n/a | RNA microarray | 43 of which 7 CUP and 6 off-panel samples | Concordance of 79% between tissue of origin test and initial histopathogenic diagnosis and further clinical and IHC workup of the disconcordant cases led to 97% agreement. For off-panel and CUP samples, tissue and cell type may be confounded by the test and careful clinicopathologic assessment is needed when interpreting results. |
| Monzon et al.^20^ | 2010 | Retrospective analysis of tissue samples | 1550 | RNA microarray | 21 | Single primary site identification in 16 of 21 (76%) specimens, indeterminate in 5 (24%). Positive results were consistent with clinicopathologic suggestions in 10 of 16 cases (62%). The remaining 6 cases were considered plausible based on clinical information. |
| Greco et al.^21^ | 2010 | Retrospective analysis of tissue samples | 87 | RT-PCR assays | 28, assay completed successfully in 20 | 15 of 20 assay predictions (75%) corresponded to actual primary sites identified after the initial diagnosis of CUP. Assay prediction was incorrect for 3 and not possible for 2 cases. Clinicopathologic findings were helpful in identifying the primary site and complement the molecular assay findings. |
| Rosenwald et al.^22^ | 2010 | Retrospective analysis of tissue samples | 48 | qRT-PCR | 204 of which 188 results could be used | For 159 of the 188 samples (85%), tissue of origin was predicted by at least one of two applied algorithms. For 124 samples (66%) the two algorithms agreed. Specificity in this group ranged averaged above 99%. |
| Morawietz et al.^23^ | 2010 | Retrospective analysis of tissue samples | 495 | RNA microarray | 24 | In 13 of 24 cases (54%) the same primary was proposed by IHC and gene expression profiling. In 7 cases there was discordance between the two and 4 cases remained unidentified. |
| van Laar et al.^24^ | 2009 | Retrospective analysis of tissue samples | 495 | RNA microarray | 13 | Analysis of 13 previously published CUP specimens yielded predicted tumor origins in line with the clinical suspicion in 12 cases (92%). |
| Monzon et al.^25^ | 2009 | Retrospective analysis of tissue samples | 1550 | RNA microarray | 547 | Gene profile results showed 87.8% overall agreement with the reference diagnosis. Performance within the subgroup of metastatic tumors was found to be slightly lower with 84.5% compared to 90.7% of the poorly differentiated and undifferentiated primary tumors. |
| Varadhachary et al.^26^ | 2008 | Retrospective analysis of tissue samples and prospective patient cohort | 10 | RT-PCR assay | Assay conducted on 104 (retrospective analysis of 78 prospective 42, total 120) | Identification of tissue of origin for 63 of 104 patients (61%), while for 41 patients (39%), the molecular profiles were not specific for the six tumor types detectable by the assay. The assigned tissue of origin was compatible with clinicopathologic features for most patients. |
| Bridgewater et al.^27^ | 2008 | Retrospective analysis of tissue samples | 495 | RNA microarray | 21 | Gene expression microarray confirmed the clinicians' suspicion in 16 out of 21 cases, while there was inconsistency with the clinical or pathological picture in 5 patients. The improved diagnoses would have influenced the management in 12 out of 21 cases. |
| Horlings et al.^28^ | 2008 | Analysis of tissue samples in parallel with standard pathologic evaluation | 495 | RNA microarray | 38 | Gene expression profiling identified 15 of 16 (94%) CUP patients whose diagnosis was solved with immunohistochemistry, and made a valuable contribution to a potential site of origin in 14 of the remaining 22 patients (64%). Misclassification of 7 of 11 lung and 3 of 3 pancreatic cancer patients. |
| Talantov et al.^29^ | 2006 | Retrospective analysis of tissue samples | 10 | qRT-PCR | 48 | Prediction of tissue of origin of metastatic carcinomas in 76% of 48 samples. |
| Tothill et al.^30^ | 2005 | Retrospective analysis of tissue samples | 79 | qPCR and microarray | 13 | Microarray classifier capable of making high confidence predictions in 11 of 13 cases. |
| Ramaswamy et al.^31^ | 2001 | Retrospective analysis of tissue samples | 16063 | oligonucleotide microarray | 54 of which 20 poorly differentiated adenocarcinomas | Overall classification accuracy was 78%. Poorly differentiated cancers resulted in low-confidence predictions and could not be accurately classified according to their tissue of origin, indicating that they are molecularly distinct entities with dramatically different gene expression. |

1. Greco FA, Hainsworth JD. Renal Cell Carcinoma Presenting as Carcinoma of Unknown Primary Site: Recognition of a Treatable Patient Subset. Clin Genitourin Cancer. 2018.

2. Moran S, Martinez-Cardus A, Sayols S, et al. Epigenetic profiling to classify cancer of unknown primary: a multicentre, retrospective analysis. Lancet Oncol. 2016;17: 1386-1395.

3. Greco FA, Lennington WJ, Spigel DR, Hainsworth JD. Poorly differentiated neoplasms of unknown primary site: diagnostic usefulness of a molecular cancer classifier assay. Mol Diagn Ther. 2015;19: 91-97.

4. Tothill RW, Shi F, Paiman L, et al. Development and validation of a gene expression tumour classifier for cancer of unknown primary. Pathology. 2015;47: 7-12.

5. Pentheroudakis G, Kotteas EA, Kotoula V, et al. Mutational profiling of the RAS, PI3K, MET and b-catenin pathways in cancer of unknown primary: a retrospective study of the Hellenic Cooperative Oncology Group. Clin Exp Metastasis. 2014;31: 761-769.

6. Kurahashi I, Fujita Y, Arao T, et al. A microarray-based gene expression analysis to identify diagnostic biomarkers for unknown primary cancer. PLoS One. 2013;8: e63249.

7. Greco FA, Lennington WJ, Spigel DR, Hainsworth JD. Molecular profiling diagnosis in unknown primary cancer: accuracy and ability to complement standard pathology. J Natl Cancer Inst. 2013;105: 782-790.

8. Hainsworth JD, Rubin MS, Spigel DR, et al. Molecular gene expression profiling to predict the tissue of origin and direct site-specific therapy in patients with carcinoma of unknown primary site: a prospective trial of the Sarah Cannon research institute. J Clin Oncol. 2013;31: 217-223.

9. Ades F, de Azambuja E, Daugaard G, et al. Comparison of a gene expression profiling strategy to standard clinical work-up for determination of tumour origin in cancer of unknown primary (CUP). J Chemother. 2013;25: 239-246.

10. Tan DS, Montoya J, Ng QS, et al. Molecular profiling for druggable genetic abnormalities in carcinoma of unknown primary. J Clin Oncol. 2013;31: e237-239.

11. Fernandez AF, Assenov Y, Martin-Subero JI, et al. A DNA methylation fingerprint of 1628 human samples. Genome Res. 2012;22: 407-419.

12. Meiri E, Mueller WC, Rosenwald S, et al. A second-generation microRNA-based assay for diagnosing tumor tissue origin. Oncologist. 2012;17: 801-812.

13. Ohta S, Cho Y, Shibata M, et al. Possibility of molecular targeting therapy for the treatment of cancer of unknown primary origin by analysis of intracellular signaling molecules. Exp Ther Med. 2012;3: 547-549.

14. Gross-Goupil M, Massard C, Lesimple T, et al. Identifying the primary site using gene expression profiling in patients with carcinoma of an unknown primary (CUP): a feasibility study from the GEFCAPI. Onkologie. 2012;35: 54-55.

15. Hainsworth JD, Schnabel CA, Erlander MG, Haines DW, 3rd, Greco FA. A retrospective study of treatment outcomes in patients with carcinoma of unknown primary site and a colorectal cancer molecular profile. Clin Colorectal Cancer. 2012;11: 112-118.

16. Sorscher SM, Greco FA. Papillary Renal Carcinoma Presenting as a Cancer of Unknown Primary (CUP) and Diagnosed through Gene Expression Profiling. Case Rep Oncol. 2012;5: 229-232.

17. Varadhachary GR, Spector Y, Abbruzzese JL, et al. Prospective gene signature study using microRNA to identify the tissue of origin in patients with carcinoma of unknown primary. Clin Cancer Res. 2011;17: 4063-4070.

18. Ferracin M, Pedriali M, Veronese A, et al. MicroRNA profiling for the identification of cancers with unknown primary tissue-of-origin. J Pathol. 2011;225: 43-53.

19. Dumur CI, Fuller CE, Blevins TL, et al. Clinical verification of the performance of the pathwork tissue of origin test: utility and limitations. Am J Clin Pathol. 2011;136: 924-933.

20. Monzon FA, Medeiros F, Lyons-Weiler M, Henner WD. Identification of tissue of origin in carcinoma of unknown primary with a microarray-based gene expression test. Diagn Pathol. 2010;5: 3.

21. Greco FA, Spigel DR, Yardley DA, Erlander MG, Ma XJ, Hainsworth JD. Molecular profiling in unknown primary cancer: accuracy of tissue of origin prediction. Oncologist. 2010;15: 500-506.

22. Rosenwald S, Gilad S, Benjamin S, et al. Validation of a microRNA-based qRT-PCR test for accurate identification of tumor tissue origin. Mod Pathol. 2010;23: 814-823.

23. Morawietz L, Floore A, Stork-Sloots L, et al. Comparison of histopathological and gene expression-based typing of cancer of unknown primary. Virchows Arch. 2010;456: 23-29.

24. van Laar RK, Ma XJ, de Jong D, et al. Implementation of a novel microarray-based diagnostic test for cancer of unknown primary. Int J Cancer. 2009;125: 1390-1397.

25. Monzon FA, Lyons-Weiler M, Buturovic LJ, et al. Multicenter validation of a 1,550-gene expression profile for identification of tumor tissue of origin. J Clin Oncol. 2009;27: 2503-2508.

26. Varadhachary GR, Talantov D, Raber MN, et al. Molecular profiling of carcinoma of unknown primary and correlation with clinical evaluation. J Clin Oncol. 2008;26: 4442-4448.

27. Bridgewater J, van Laar R, Floore A, Van TVL. Gene expression profiling may improve diagnosis in patients with carcinoma of unknown primary. Br J Cancer. 2008;98: 1425-1430.

28. Horlings HM, van Laar RK, Kerst JM, et al. Gene expression profiling to identify the histogenetic origin of metastatic adenocarcinomas of unknown primary. J Clin Oncol. 2008;26: 4435-4441.

29. Talantov D, Baden J, Jatkoe T, et al. A quantitative reverse transcriptase-polymerase chain reaction assay to identify metastatic carcinoma tissue of origin. J Mol Diagn. 2006;8: 320-329.

30. Tothill RW, Kowalczyk A, Rischin D, et al. An expression-based site of origin diagnostic method designed for clinical application to cancer of unknown origin. Cancer Res. 2005;65: 4031-4040.

31. Ramaswamy S, Tamayo P, Rifkin R, et al. Multiclass cancer diagnosis using tumor gene expression signatures. Proc Natl Acad Sci U S A. 2001;98: 15149-15154.
