# Supplementary material for: Cancer of unknown primary—Epidemiological trends and relevance of comprehensive genomic profiling
Source: Cancer Med. 2018 Jul 17;7(9):4814–24. doi: 10.1002/cam4.1689 (PMC6144156; doi:10.1002/cam4.1689)
Supplement: Supplementary file 1 [file CAM4-7-4814-s001.pdf]

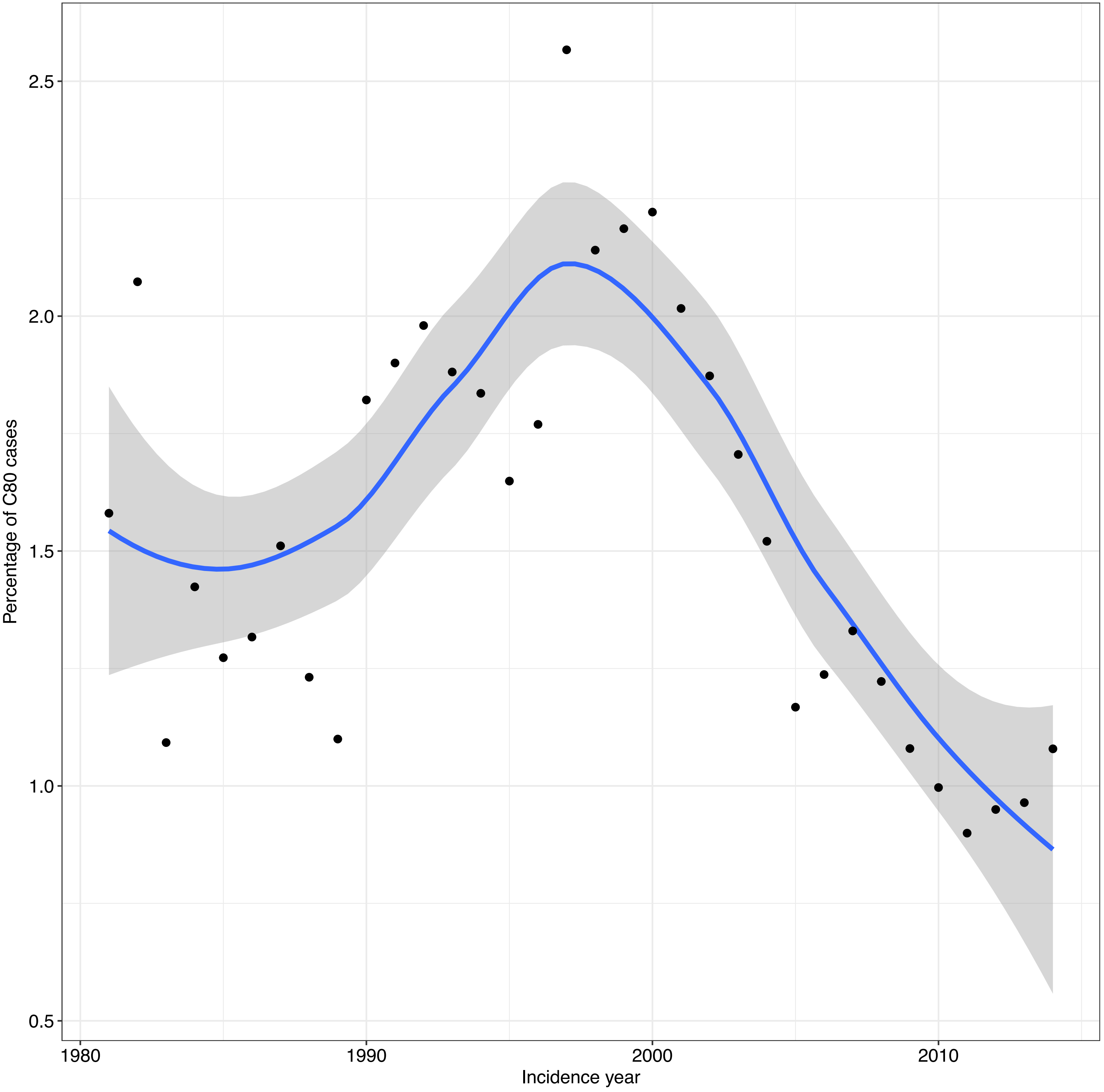

Elfi t qd R09BTO b` r dr ` r odqbdms` f d nemdv b` mbdq b` r dr ch f mnr dc hmsgd b` msnmneYt qhbg 0870,1/ 03-
